# Extended Medicaid coverage will improve access but insufficient to enhance postpartum care utilization: a secondary analysis of the 2016–2019 Arizona Medicaid claims

**DOI:** 10.3389/fpubh.2023.1281574

**Published:** 2024-01-08

**Authors:** Abidemi Okechukwu, Ivo Abraham, Chinedu Okechukwu, Priscilla Magrath, David G. Marrero, Leslie V. Farland, Halimatou Alaofe

**Affiliations:** ^1^Mel and Enid Zuckerman College of Public Health, University of Arizona, Tucson, AZ, United States; ^2^R. Ken Colt College of Pharmacy, University of Arizona, Tucson, AZ, United States; ^3^Department of Business Analytics, Eller College of Management, University of Arizona, Tucson, AZ, United States; ^4^Center for Health Disparities Research, University of Arizona Health Sciences (UAHS), Tucson, AZ, United States

**Keywords:** postpartum, low-income insurance, healthcare utilization, Medicaid extension, maternal health

## Abstract

**Introduction:**

Postpartum Medicaid eligibility extensions may increase access to healthcare for low-income women. However, its implications for healthcare utilization are unknown.

**Methods:**

We analyzed the linked-infant birth certificate and claims data of women whose childbirths were paid for by Medicaid between 2016 and 2019 in Arizona, United States. We evaluated associations between postpartum care visits and Medicaid insurance type and assessed effect modification by the delivery route and type of residence.

**Results:**

Women with pregnancy-related Medicaid insurance were less likely to attend postpartum visits, with an adjusted odds ratio (aOR) of 0.70 and a 95% confidence interval (CI) of 0.66 to 0.74 than those with continuous Medicaid insurance. Younger age, rural residence [aOR 0.83, CI 0.78, 0.88], vaginal delivery route [aOR 0.11, CI 0.10, 0.12], and the absence of complications during/after childbirth [aOR 0.58, CI 0.49, 0.70] were associated with the absence of postpartum care visit. Low-income women who lost their pregnancy-related Medicaid coverage after 60 days in Arizona experienced lower rates of postpartum care utilization.

**Discussion:**

Interventions to improve postpartum utilization should be considered beyond extending postpartum Medicaid coverage for low-income women.

## Introduction

Health agencies worldwide are expanding their focus to ensure that birthing women and their babies do not merely survive the process of childbirth but also thrive and reach their potential for health and well-being (1–3). The postpartum is a critical period for women and newborns. It determines long-term women’s health and well-being because the availability and quality of care and support provided during postpartum enhance preconception and inter-conception health (4).

The American Congress of Obstetricians and Gynecologists (ACOG) recommends that postpartum care be ongoing and tailored to meet the individual needs of women. ACOG also notes that optimized postpartum care should provide an initial assessment for women and ongoing care as needed. Ongoing care should include comprehensive well-woman visits, counseling and contraception services, and managing preconception comorbidities through coordinated care that should extend up to 1 year after childbirth (5). Postpartum healthcare supports women’s health by screening and assessing physical, mental, and social health risks (1). This care also provides opportunities to prevent, diagnose, and treat complications from pregnancy and childbirth, manage preconception health issues such as hypertension and diabetes, provide contraception and family planning services, facilitate community support, and address other social needs. Given the opportunities that postpartum care offers, improving women’s access to high-quality postpartum care becomes a priority for policy implementation.

Despite the many benefits of postpartum healthcare and recommendations from various health agendas, many women do not have access to care or fail to utilize available healthcare during postpartum. Over 40% of childbearing women in the United States (U.S.) do not attend postpartum care (6, 7). Several studies have shown that access to postpartum care primarily depends on health insurance (8–10). Furthermore, postpartum care utilization is contingent on affordability and social determinants of health, such as place of residence, availability of transportation, and childcare services that facilitate care utilization (11–13). As such, women who belong to disparity populations, including low-income, rural, and racial minorities, may be disproportionately affected by inequities in access and use of postpartum care, increasing the disparities in the risk for adverse maternal outcomes and deaths (14).

Medicaid, a United States federal and state-funded program, provides health coverage to eligible low-income adults. In 2019, Medicaid was the payment source for 42% of births in the United States (15). Low-income women who do not qualify for continuous adult Medicaid insurance may gain eligibility for pregnancy-related Medicaid insurance that covers medical care during pregnancy and up to 60 days after childbirth. While pregnancy-related Medicaid insurance provides critical support for low-income women who earn above the threshold for continuous adult insurance, access to care is short-lived since this coverage expires after 60 days postpartum. This coverage disruption or loss reduces access to routine and *ad hoc* medical services from Day 61 to Day 365 (1 year) after birth (16). Extending Medicaid postpartum coverage through the first year after childbirth will increase access to care for beneficiaries of pregnancy-related insurance (17, 18). Several federal and state initiatives are implementing policies to improve access to healthcare during postpartum (4, 5, 19, 20). More recently, the 2021 American Rescue Plan Act (ARP) provides a pathway for states to extend postpartum insurance through an 1,115 waiver or a State Plan Amendment under the Medicaid and Child Health Insurance (CHIP) plans (19).

The Arizona Medicaid Agency, known as the Arizona Health Care Cost Containment System (AHCCCS), provides continuous medical assistance to low-income women with a household income at and below 133% federal poverty level (FPL) and pregnancy-related insurance for women with household income between 133 and 156% FPL (21). AHCCCS pays for about 50% of births in Arizona (22). In 2022, AHCCCS approved extending Medicaid pregnancy-related insurance eligibility up to 1 year after childbirth through federal matching funds provided by the 2021 American Rescue Plan Act. The state allocated $2.7 million to offer health insurance coverage to eligible low-income women with household income 133–156% FPL (23). The plan to extend postpartum coverage of pregnancy-related insurance raises several questions: How will this policy impact federal and state budgets? How will it improve postpartum care utilization and women’s health outcomes, particularly among populations that experience excess health disparities? Because Medicaid policies vary by state, a granular understanding of baseline postpartum care utilization is crucial to implementing Arizona’s new Medicaid (AHCCCS) policy. Thus, this study aims to describe insurance coverage patterns and postpartum care utilization for women whose pregnancy care and childbirths were paid for by AHCCCS and to investigate factors associated with postpartum healthcare utilization among this cohort. This analysis will contribute important baseline information to guide health financing and policy implementation for maternal and child health in the United States.

## Materials and methods

### Study design and data source

This study is a secondary analysis of deidentified Arizona Medicaid claims data to assess the association between Medicaid insurance type and postpartum healthcare utilization and the effects of other individual and structural factors. The unit of observation was mothers of live births between 2016 and 2019 in Arizona. We obtained claims data files for women whose childbirth was paid for by AHCCCS between January 1, 2016, and December 31, 2019, from the Arizona State University Center for Health Information Research, managed on behalf of Arizona. The data included information on women’s demographics, Medicaid insurance eligibility, and health care claims, including outpatient and inpatient claims and diagnoses. Women’s demographic, eligibility, and claims information were linked to the Arizona infant birth records and women’s hospital discharge data from the Arizona Department of Health and Services. All the data files met the Health Insurance Portability and Accountability Act’s definition of a limited dataset. The study was approved by the University of Arizona Institutional Review Board (IRB_STUDY#00000208) and was exempted from further review. This study followed the Strengthening the Reporting of Observational Studies in Epidemiology (STROBE) reporting guideline for cohort studies.

### Study sample

We linked infant birth data and women’s hospital discharge data, demographics, Medicaid insurance eligibility, diagnoses, and claims files to identify a cohort of women aged 18–49 years whose delivery was paid for by AHCCCS. Included in the cohort were women who gave birth to singleton newborns in Arizona between January 1, 2016, and December 31, 2019. For women with more than one live birth during this period, we selected the most recent birth to be included in the cohort. We excluded women who needed emergency delivery services but were ineligible for Medicaid pregnancy-related insurance and women who were incarcerated at childbirth whose pregnancy care and childbirth were covered by the correctional healthcare service agency (21).

### Exposure variable

The primary exposure variable was the type of AHCCCS insurance: Medicaid pregnancy-related, which expires 60 days after childbirth, or continuous Medicaid insurance, which is continuous medical assistance for low-income adults and does not expire based on pregnancy status. To determine the enrollment period after pregnancy under AHCCCS insurance, we used eligibility beginning and ending dates to define a period of eligibility between 6 months before and 12 months after childbirth. For each woman in the study sample, the follow-up period was defined as 365 days after the infant’s date of birth. Subsequently, we categorized women’s insurance types into continuous and pregnancy-related insurance using two criteria (Supplementary Table 1.0). First, by subtracting the end date of insurance enrollment from her infant’s birth date to evaluate the eligibility period, and second, by confirming insurance type by the enrollment category codes (24).

### Outcome variable

The outcome of interest was postpartum care utilization. We defined postpartum care utilization as a woman’s use of healthcare for routine medical and gynecological services billed as claims for postpartum encounters to Medicaid within the first year after childbirth according to the CDC vital statistics reporting guidance and existing literature on the postpartum period (25, 26). Postpartum encounters were identified by linking the AHCCCS claims file with the diagnosis files within the one-year postpartum period. To examine the types of postpartum care and evaluate women’s use of healthcare services after childbirth, we adapted the 2021 HEDIS Documentation and Coding Guideline definition and codes for postpartum care. We used the International Classification of Disease Codes (ICD-10), the Health Care Common Procedure Coding System (HCPCS), and Current Procedural Terminology (CPT) codes from the 2021 HEDIS guideline to categorize postpartum care into three types: care for birth complications, cervical cytology, and postpartum visits (Supplementary Table 2.0), (27).

The indicator for postpartum care utilization was a binary variable: the presence or absence of at least one postpartum care visit. For sensitivity analysis, we also computed the frequency of postpartum care visits (0, 1, 2, 3, 4, 5, and > 5 visits within the first 365 days after childbirth). First, we estimated the start date of each type of postpartum care from the claims using infants’ birth dates to calculate the start date of postpartum for each associated claim. Subsequently, we determined the timing of each postpartum visit in days postpartum by deducting the infant’s birth date from the service date on the claims. This action created a longitudinal claims dataset for women in the study cohort. We then determined the frequency of postpartum visits by counting the number of visits for each woman in the cohort and recategorized visits into a binary postpartum visit variable (yes/no).

### Covariates

We extracted information on women’s demography and birthing outcomes from the infants’ birth and women’s hospital discharge records. The following covariates were selected based on the literature on covariates of postpartum care utilization (6, 13). Residential zip codes were linked to the USDA Rural–Urban Commuting Area (RUCA) crosswalk (28) to categorize them into rural and urban residences according to the Health Resources and Services Administration’s RUCA categorization of 1–4 as rural and 5–10 as urban areas (29). Categories of maternal age at childbirth (< 20, 20–29, 30–39, and 40–49), race/ethnicity (Asian, Black/African American, Hispanic, Native American, Other, Pacific Islander, and White), type of residence (rural or urban), prenatal care attendance (yes or no), type of delivery facility (hospital or other), route of delivery (vaginal or cesarean section), and maternal complications at childbirth based on infant record (yes or no) were also included as covariates in our analyses.

### Statistical analysis

We assessed the distribution of maternal characteristics by insurance type, and chi-square tests were used to compare these characteristics between women with AHCCCS pregnancy-related insurance and AHCCCS continuous insurance. We analyzed the association between insurance type and postpartum care visits in two models using logistic regression analysis that yielded odds ratios (OR) and 95% confidence intervals (CI). Model 1 evaluated the independent relationship between postpartum utilization with insurance type and covariates. Model 2 was an adjusted multivariable logistic regression analysis and incorporated interaction terms between insurance type and two covariates, route of delivery, and type of residence, to evaluate potential effect modification. We evaluated model fit using the Hosmer-Lemeshow goodness-of-fit test and c-statistic to assess model discrimination.

For sensitivity analysis, we utilized the frequency of visits variable (0, 1, 2, 3, 4, 5, and >5) and conducted Poisson regression analysis with robust error variance using a generalized linear model to evaluate the association between insurance type and the frequency variable of postpartum visits and covariates. Data files were read, merged, and cleaned using RStudio statistical software, and all statistical analyses were performed with SAS Version 9.4 (SAS Institute: Cary, NC, United States). We set all statistical tests and 95% confidence intervals (CI) with a two-tailed value of *p* less than 0.05 as statistically significant.

## Results

**Figure 1:**
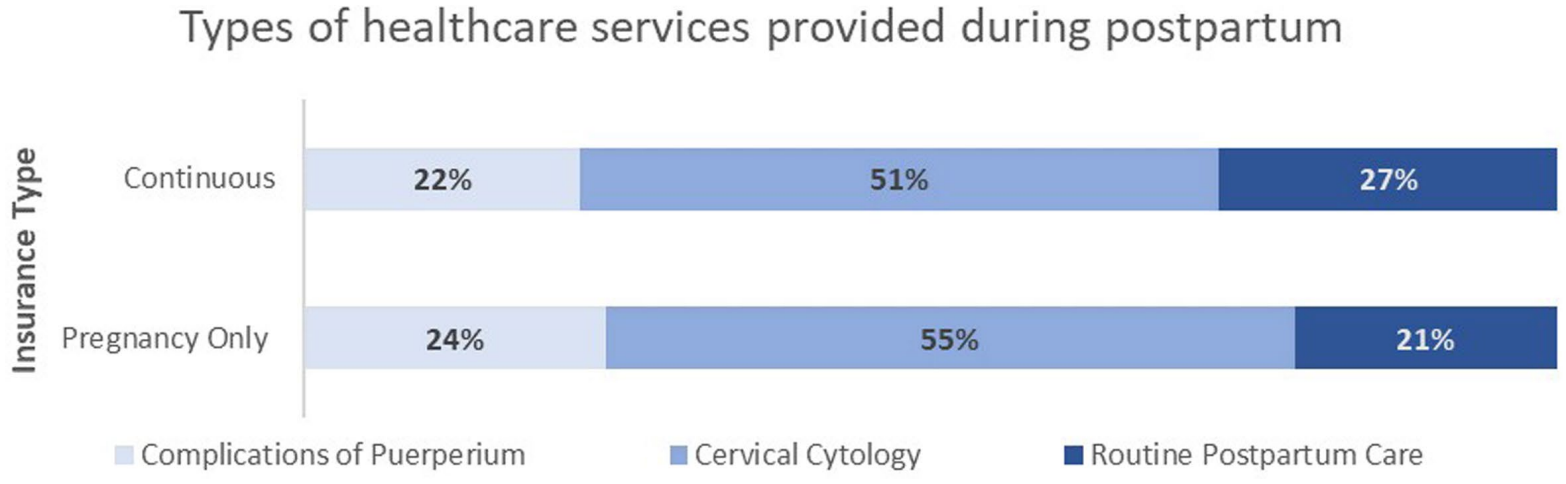
Type of services received by women in the cohort during postpartum.

Having received insurance from AHCCCS between 2016 and 2019, 58,500 women who met the inclusion criteria for the cohort analysis were identified. Of the 124,513 claims linked with this cohort, 51% were billing for services for cervical cytology, 21% for labor and delivery complications services, and 28% for routine postpartum services (Figure 1). The types of health care services utilized during postpartum did not differ by type of insurance.

**Figure 2:**
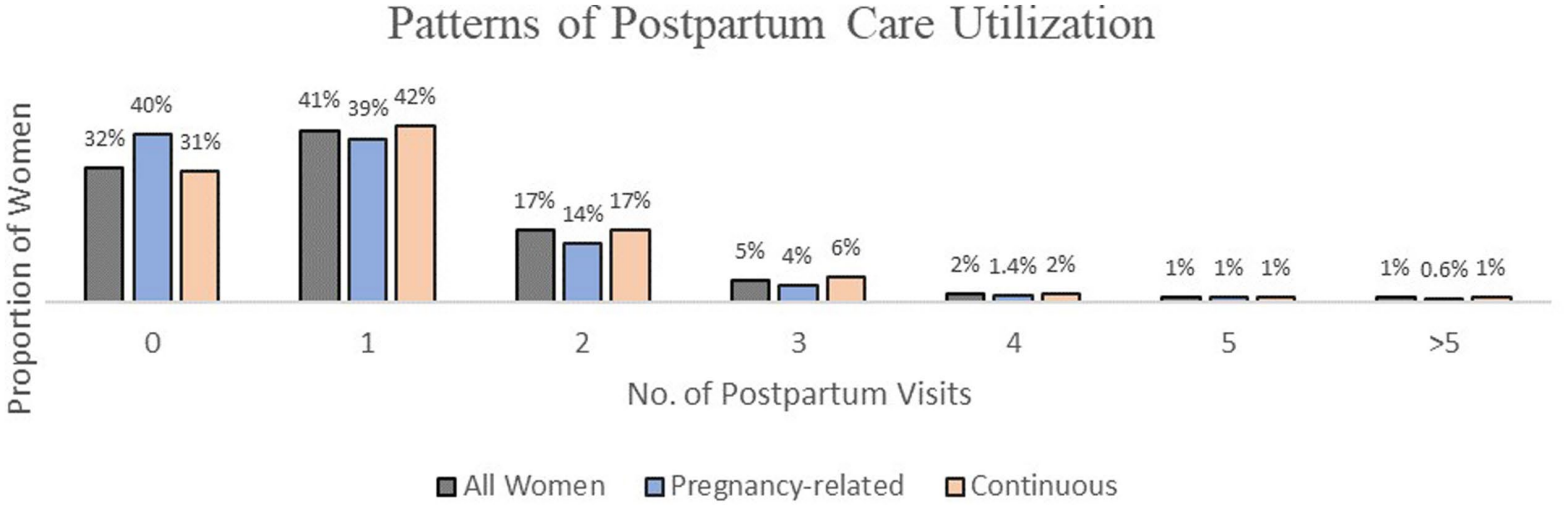
Patterns of postpartum care utilization in the cohort.

The pregnancy-related AHCCCS group had a higher percentage of women between 18-20 years old (11% vs. 5%) and a lower proportion of women between 30 to 39 years (25% vs. 29%) compared to the continuous AHCCCS group. Both groups of women had similar proportions for race and ethnicity, U.S. citizenship, residence, delivery facility, and complications following childbirth. Women in the pregnancy-related AHCCCS group were less likely to attend prenatal visits (94% vs. 97%), use tobacco (16% vs. 18%), and participate in postpartum visits (60% vs. 69%) as compared to the continuous AHCCCS group. In the analytic cohort sample of 58,500 women, 38,628 (68%) attended at least one postpartum care visit, when stratified by insurance type, more of the continuous AHCCCS group were likely to participate in at least one postpartum visit (42% vs. 39%), attend two visits (17% vs. 14%), and attend three visits (6% vs. 4%) than the pregnancy-related AHCCCS group. In addition, women in the pregnancy-related group were more likely to have no postpartum visits in the first year after childbirth (40%) compared to the continuous insurance group (31%) (Figure 2).

Table 1 reports the characteristics of women aged 18 - 49 with singleton births whose childbirths and postpartum care were paid for by AHCCCS, grouped by continuous and pregnancy-related insurance categories as provided. Six thousand four hundred and thirty-eight (6,438) women had pregnancy-related insurance (11%), and 52,062 had continuous insurance (89%). Of all women in the cohort, 1,170 were Asian (2%), 6,677 African American (11.4%), 6,346 Hispanic (10.8%), 3,206 American Indian & Alaskan Native (5.5%), 287 Pacific Islander (0.5%) and 38,7768 White (66%) individuals; Other included 2,046 (3.5%) individuals who identified as mixed race or ethnicity. Fifty-five thousand, five hundred and eighty-one (55,581) women were U.S. citizens (89%). The majority, 52,447 (90%), lived in urban areas in Arizona at the time of birth of their infants, and 10,644 (18%) reported using tobacco before pregnancy. Over 99% of women had their deliveries at a hospital, 97% had at least one prenatal visit before childbirth, 46,519 (80%) gave birth through vaginal delivery, and 782 (1%) had complications immediately following childbirth.

**Table 1:** Distribution of maternal characteristics and outcomes by AHCCCS insurance type.

	Participants, No. (%)			
		Insurance type		
Characteristics	All women	Pregnancy-related	Continuous	Chi-square
	*N* = 58,500	*N* = 6,438	*N* = 52,062	*p* value
*Age*				<0.0001
18–20	3,363 (6)	677 (11)	2,686 (5)	
21–29	37,344 (64)	4,016 (62)	33,328 (64)	
30–39	16,469 (28)	1,608 (25)	14,861 (29)	
40–49	1,324 (2)	137 (2)	1,187 (2)	
*Race/Ethnicity*				<0.0001
Asian	1,170 (2)	126 (2)	1,044 (2)	
Black/African American	6,677 (11)	654 (10)	6,023 (12)	
Hispanic	6,346 (11)	863 (13)	5,483 (11)	
Native American	3,206 (6)	406 (6)	2,800 (5)	
Other^a^	2046 (3.5)	306 (5)	1,737 (3)	
Pacific Islander	287 (0.5)	39 (1)	251 (0.5)	
White	38,768 (66)	4,044 (63)	34,724 (66.5)	
*Citizenship*				0.023
US Citizen	55,581 (95)	6,076 (94)	49,505 (95)	
Other^b^	2,919 (5)	358 (6)	2,561 (5)	
*Type of residence*				0.313
Rural	6,053 (10)	646 (10)	5,407 (10)	
Urban	52,447 (90)	5,792 (90)	46,655 (90)	
*Delivery facility*				0.8238
Hospital	58,417 (99.9)	6,430 (99.9)	51,987 (99.9)	
Other^c^	83 (0.1)	8 (0.1)	75 (0.1)	
*Prenatal visit*				0.0001
Yes	56,885 (97)	6,212 (94)	50,673 (97)	
No	1,615 (3)	226 (4)	1,389 (3)	
*Route of delivery*				<0.0001
Vaginal delivery	46,519 (80)	5,259 (82)	41,260 (79)	
Cesarean section	11,981 (20)	1,179 (18)	10,802 (21)	
*Complications during/after childbirth*				0.4588
Yes	782 (1)	93 (1)	689 (1)	
No	57,811 (99)	6,438 (99)	51,373 (99)	
*Tobacco use*				0.0002
Yes	10,644 (18)	1,062 (16)	9,582 (18)	
No	47,856 (82)	5,376 (84)	42,480 (82)	
*Any postpartum visit*				<0.0001
Yes	39,628 (68)	3,871 (60)	35,757 (69)	
No	18,872 (32)	2,567 (40)	16,305 (31)	
*No. of postpartum visits*				<0.0001
0	18,872 (32)	2,567 (40)	16,305 (31)	
1	24,337 (41)	2,533 (39)	21,804 (42)	
2	9,954 (17)	910 (14)	9,044 (17)	
3	3,256 (5)	246 (4)	3,010 (6)	
4	1,096 (2)	93 (1.4)	1,003 (2)	
5	442 (1)	51 (1)	391 (1)	
>5	543 (1)	38 (0.6)	505 (1)	

^a^Women who identified as multiple races or ethnicities. ^b^Non-US citizens or permanent residents. ^c^Facilities different from a hospital, e.g., physicians’ clinics, birthing centers, and residential facilities.

Based on logistic regression analysis Model 1, women with pregnancy-related insurance had lower odds of attending postpartum visits, OR 0.69, 95% CI: 0.65, 0.73 than those with continuous insurance (Table 2). Postpartum visits were associated with rural residence [OR: 0.84, 95% CI: 0.80, 0.89], prenatal visit attendance [OR: 1.63, 95% CI: 1.48, 1.81], delivery route [OR: 0.11, 95% CI: 0.10, 0.12], and complications during or after childbirth [OR: 1.93, 95% CI: 1.61, 2.30]. In the multivariable logistic regression Model 2, we observed that women with pregnancy-related insurance had lower odds of attending postpartum visits [Adjusted Odds Ratio (aOR: 0.70, 95% CI: 0.66, 0.74] than those with continuous insurance. Compared to women younger than 20 years, older age was associated with 20% - 31% higher odds of a postpartum visit. At the same time, rural residents had 17% lower odds of a postpartum visit than urban residents [aOR: 0.83, 95% CI: 0.78, 0.88], and prenatal visit attendance was associated with higher odds of a postpartum visit [aOR: 1.73, 95% CI: 1.43, 1.92]. Compared to women who delivered their babies through cesarean section, those who delivered through the vaginal route had 89% lesser odds of a postpartum visit [aOR: 0.11, 95% CI: 0.10, 0.12] meanwhile, women with no complication after childbirth had 52% lower odds of a postpartum visit [aOR: 0.58, 95% CI: 0.49, 0.70] (Table 2). The route of delivery (cesarean or vaginal birth) and type of residence (rural or urban) did not modify the relationship between postpartum visit and insurance type (Supplementary Table 3.0). A sensitivity analysis that tested the association between postpartum visits and frequency (counts) of postpartum care visits as the outcome variable in a multivariable Poisson regression model was congruent with our primary research. Women with pregnancy-related AHCCCS were less likely to have more postpartum visits, incident risk ratio (IRR) was 0.85 (Supplementary Table 4.0). The logistic regression models for the primary analysis (Models 1 & 2) were assumed to be independent observations. The C-statistic was 0.65, and the *p*-values for the Hosmer and Lemeshow test of goodness of fit for the adjusted model was 0.2719 (Supplementary Tables 5.0.A,B).

**Table 2:** Logistic regression model of factors associated with postpartum care visit.

	Model 1	Model 2^*^
Characteristic	OR (95% CI)	aOR (95% CI)
*Insurance type*		
Pregnancy-related	0.69 (0.65, 0.73)	0.70 (0.66, 0.74)
Continuous	1 [Reference]	1 [Reference]
*Age*		
21–29	1.34 (1.24, 1.44)	1.20 (1.11, 1.29)
30–39	1.68 (1.56, 1.81)	1.31 (1.21, 1.42)
40–49	1.85 (1.60, 2.12)	1.28 (1.10, 1.48)
18–20	1 [Reference]	1 [Reference]
*Race/Ethnicity*		
Asian	1.15 (1.01, 1.30)	1.11 (0.97, 1.27)
Black/African American	1.06 (1.01, 1.13)	0.98 (0.93, 1.04)
Hispanic	0.94 (0.89, 0.99)	0.95 (0.90, 1.01)
Native American	1.14 (1.03, 1.26)	1.17 (1.06, 1.30)
Other	0.99 (0.77, 1.27)	0.98 (0.76, 1.27)
Pacific Islander	0.84 (0.78, 0.91)	0.87 (0.80, 0.94)
White	1 [Reference]	1 [Reference]
*Type of residence*		
Rural	0.84 (0.80, 0.89)	0.83 (0.78, 0.88)
Urban	1 [Reference]	1 [Reference]
*Prenatal visit*		
Yes	1.63 (1.48, 1.81)	1.73 (1.55, 1.92)
No	1 [Reference]	1 [Reference]
*Route of delivery*		
Vaginal	0.11 (0.10, 0.12)	0.11 (0.10, 0.12)
Cesarean	1 [Reference]	1 [Reference]
*Complications during/after childbirth*		
No	0.52 (0.44, 0.62)	0.58 (0.49, 0.70)
Yes	1 [Reference]	1 [Reference]

^*^Model 2 was adjusted for age, race, type of residence, prenatal visit, route of delivery, and complication during or after childbirth.

OR, Odds ratio; Ref, Reference; aOR, Adjusted odds ratio; CI, Confidence interval.

## Discussion

We observed statistically significant differences in postpartum care utilization by AHCCS insurance type, rurality of residence, history of prenatal visits during pregnancy, the delivery route, and complications during or after childbirth. Overall, Arizona women with pregnancy-related Medicaid insurance were less likely to attend any postpartum care than their counterparts with continuous eligibility for low-income adult Medicaid insurance. This relationship remained statistically significant after adjusting for differences in individual characteristics and events during pregnancy and childbirth. We observed patterns of postpartum care utilization similar to those in prior studies (18, 30, 31). Women on pregnancy-related Medicaid insurance are generally understood to be prone to insurance transitions or loss between 60 and 90 days after childbirth, resulting in inadequate or no postpartum care (16, 18). While it is assumed these women could afford commercial insurance after delivery because they come from low-income households with higher income levels than those with continuous adult coverage, many still experience disruptions or total loss of coverage that reduce their access to all forms of postpartum care. Comparisons between the two groups of Medicaid-financed insurance in Wisconsin reported that women with pregnancy-related insurance were younger, and prenatal visit attendance was the strongest predictor of postpartum visits (30). Comparing our study to the Wisconsin study, the most robust correlates in our analysis were the delivery route and complications during or after childbirth.

A study on postpartum care utilization in Illinois found that utilization differed by race, ethnicity, and rural residence (31). In our research, rural residents had a lower likelihood of postpartum visits than their urban counterparts. Still, compared to the Illinois study, postpartum care utilization did not vary by race or ethnicity. ACOG’s recommendations for postpartum care imply value-based care that centers on the individual needs of the women and the quality of care. Leaving some latitude for flexibility with standards of care, ACOG urges postpartum care to be continuous and coordinated (5). Our study findings did not suggest any form of routine or coordinated postpartum visits. Postpartum visits appeared isolated from routine clinical visits. Like Rankin et al. (31), we found that women utilized healthcare services during postpartum but not primarily for routine postpartum care. In our analysis, over 51% of claims were for cervical cytology and only 25% for routine postpartum care. This loosely implies that providing insurance is necessary for access to care but insufficient for the actual utilization of services. Given the low postpartum utilization rates irrespective of the type or duration of insurance, we can infer that women are not taking advantage of the opportunity that ensures continuity of care during postpartum. Hence, interventions must tackle all the barriers to postpartum care utilization.

Nearly 90% of the population in Arizona is urban, concentrated in two counties flanked by anchoring cities along the United States. Mexico border (32). With a substantial population of racial minority groups (Latinx and Native Americans) and a fiscally conservative state legislature, our study provides compelling findings about postpartum care utilization for populations with excess disparities in maternal outcomes. Our dataset was comprised of nearly all low-income childbearing women in Arizona for 4 years, assuming that Medicaid covered them. With Medicaid paying for about 50% of maternity care in Arizona, our comprehensive and diversified analytical sample was representative of low-income birthing women in Arizona (33). We applied the standardized HEDIS definitions and ICD-10 codes for postpartum care to characterize medical services provided to women after childbirth, making it possible for other researchers to replicate our definitions in different regional and state datasets. Our analysis explored the effect of other categorizations of disparity populations beyond low income, notably rural residence, as well as race and ethnic minority, which have strong relevance to Arizona’s population and demography.

Our data analysis also had some limitations. First, the AHCCCS Medicaid claims data did not contain information on women’s education, income, contraception care, behavioral health services, or *ad hoc* services requiring postpartum visits. AHCCCS does not pay for postpartum family planning services under pregnancy-related insurance for women, and behavioral health services are funded under another type of state-level policy (34). These issues raised concerns about data quality and prevented sub-analyses that could have highlighted other disparities in care utilization. Second, we could not evaluate the role of social determinants of health (SDOH), such as transportation and childcare services, because the data needed to contain related SDOH variables.

We could not ascertain if women who lost coverage gained commercial or other forms of insurance. Managing duplicate claims from multiple types of providers on the same service date was a challenge and required that we make assumptions, and we would expect some non-differential misclassification (35). The quality of postpartum visits or information on women’s satisfaction with care should be evaluated since Medicaid pays for about 50% of childbirths in Arizona (22). Future research must determine postpartum care utilization among women who had miscarriages early in pregnancy, stillbirth, or neonatal deaths because the motivations and reasons for postpartum care may differ from those of women without health insurance.

### Policy implications

The findings from this study point to critical policy implications. Our findings of low postpartum utilization among all AHCCCS beneficiaries create the imperative for interventions to promote the demand and use of postpartum care. These interventions must accompany the policy that extends insurance coverage throughout the first year after delivery because the determinants of postpartum utilization are complex and require multifaceted approaches. Second, from the supply perspective, policy interventions must involve all stakeholders, including facility-based and community-based providers. Extending insurance to improve access to care for women relies on care transitions that allow for continuity of care. Care providers, particularly obstetricians, must prioritize referrals for women to their primary care providers for follow-up care after the immediate postpartum period (42 days after birth) (36). Policy solutions facilitating smooth care transitions during postpartum toward care providers are critical to the success of the postpartum coverage extension policy.

Low-income childbearing women who live in rural communities or underprivileged urban areas are often socially isolated and may have limited geographical access to postpartum care despite insurance coverage. Community-based models of care may improve access to essential medical and support services for these women. Therefore, community-based systems of care must be integrated into solutions for access and continuity of care (37). Additionally, social determinants of health, such as transportation and childcare services, play critical roles in postpartum care utilization, and rural residents may need assistance with services that facilitate care utilization (37). Rural residents may need assistance with services that facilitate utilization. Reimbursements for community-based services such as doula services can improve access and use of postpartum services and reduce disparities in adverse maternal outcomes.

## Conclusion

We found that low-income women who lost their pregnancy-related Medicaid coverage after 60 days in Arizona experienced lower rates of postpartum care utilization. Among women who lost coverage after 60 days postpartum, rural residence, absence of prenatal visits during pregnancy, childbirth through vaginal delivery, and having no complications during or after childbirth reduced the likelihood of postpartum care utilization. This supports the evidence that several supply and demand factors beyond access to health insurance influence healthcare utilization. These findings have important implications for implementing Medicaid postpartum policy; an effective policy rollout for low-income women must target nuanced determinants of postpartum utilization among rural residents in Arizona.

## Data availability statement

The data analyzed in this study is subject to the following licenses/restrictions: the dataset can only be obtained from the Arizona Department of Health Services. Requests to access these datasets should be directed to https://www.azdhs.gov/licensing/vital-records/index.php.

## Author contributions

AO: Conceptualization, Data curation, Formal analysis, Investigation, Methodology, Software, Validation, Visualization, Writing – original draft, Writing – review & editing. IA: Supervision, Writing – review & editing. CO: Data curation, Formal analysis, Methodology, Software, Validation, Writing – review & editing. PM: Writing – review & editing. DM: Writing – review & editing. LF: Writing – review & editing. HA: Project administration, Resources, Supervision, Writing – review & editing, Validation.

## References

[1] World Health Organization (2022). Special Programme of research D. WHO recommendations on maternal and newborn care for a positive postnatal experience. World Health Organization. 224 35467813

[2] Disparities (2020). Healthy people. Available at: https://www.healthypeople.gov/2020/about/foundation-health-measures/Disparities

[3] National Advisory Committee on Rural Health and Human Services (2020). Maternal and obstetric care challenges in rural AMERICA policy brief and recommendations to the secretary. Available at: https://www.hrsa.gov/sites/default/files/hrsa/advisory-committees/rural/2020-maternal-obstetric-care-challenges.pdf

[4] McCloskey L Bernstein J The Bridging the Chasm Collaborative Amutah-Onukagha N Anthony J Barger M. Bridging the chasm between pregnancy and health over the life course: a National Agenda for research and action. Womens Health Issues. (2021) 31:204–18. doi: 10.1016/j.whi.2021.01.002, PMID: 33707142 PMC8154664

[5] Auguste T Gulati M. Recommendations and conclusions presidential task force on redefining the postpartum visit Committee on obstetric practice optimizing postpartum care committee opinion optimizing postpartum care. Obstet Gynecol. (2018) 131:e140–50. doi: 10.1097/AOG.0000000000002633 29683911

[6] Bryant A Blake-Lamb T Hatoum I Kotelchuck M. Women’s use of health Care in the First 2 years postpartum: occurrence and correlates. Matern Child Health J. (2016) 20:81–91. doi: 10.1007/s10995-016-2168-9, PMID: 27502197

[7] Bennett WL Chang HY Levine DM Wang L Neale D Werner EF. Utilization of primary and obstetric care after medically complicated pregnancies: an analysis of medical claims data. J Gen Intern Med. (2014) 29:636–45. doi: 10.1007/s11606-013-2744-2 24474651 PMC3965743

[8] Dibari JN Yu SM Chao SM Lu MC. Use of postpartum care: predictors and barriers. J Pregnancy. (2014) 2014:1–8. doi: 10.1155/2014/530769 PMC3945081 24693433

[9] Henderson V Stumbras K Caskey R Haider S Rankin K Handler A. Understanding factors associated with postpartum visit attendance and contraception choices: listening to low-income postpartum women and health care providers. Matern Child Health J. (2016) 20:132–43. doi: 10.1007/s10995-016-2044-7, PMID: 27342600 PMC5290059

[10] Gordon SH Sommers BD Wilson IB Trivedi AN. Effects of medicaid expansion on postpartum coverage and outpatient utilization. Health Aff. (2020) 39:77–84. doi: 10.1377/hlthaff.2019.00547, PMID: 31905073 PMC7926836

[11] Wilcox A Levi EE Garrett JM. Predictors of non-attendance to the postpartum follow-up visit. Matern Child Health J. (2016) 20:22–7. doi: 10.1007/s10995-016-2184-9 27562797

[12] Geissler K Ranchoff BL Cooper MI Attanasio LB. Association of Insurance Status with Provision of recommended services during comprehensive postpartum visits. JAMA Netw Open. (2020) 3:e2025095–5. doi: 10.1001/jamanetworkopen.2020.25095, PMID: 33170263 PMC7656283

[13] Weir S Posner HE Zhang J Willis G Baxter JD Clark RE. Predictors of prenatal and postpartum care adequacy in a Medicaid managed care population. Womens Health Issues. (2011) 21:277–85. doi: 10.1016/j.whi.2011.03.001, PMID: 21565526

[14] Thiel de Bocanegra H Braughton M Bradsberry M Howell M Logan J Schwarz EB. Racial and ethnic disparities in postpartum care and contraception in California’s Medicaid program. Am J Obstet Gynecol. (2017) 217:47.e1–7. doi: 10.1016/j.ajog.2017.02.040, PMID: 28263752

[15] Martin JA Hamilton BE Osterman MJK (2019). Key findings data from the National Vital Statistics System. Available at: https://www.cdc.gov/nchs/products/index.htm

[16] Gordon SH Hoagland A Admon LK Daw JR. Comparison of postpartum health care use and spending among individuals with medicaid-paid births enrolled in continuous medicaid vs commercial insurance. JAMA Netw Open. (2022) 5:e223058–8. doi: 10.1001/jamanetworkopen.2022.3058, PMID: 35302626 PMC8933732

[17] Daw JR Winkelman TNA Dalton VK Kozhimannil KB Admon LK. Medicaid expansion improved perinatal insurance continuity for low-income women. Health Aff. (2020) 39:1531–9. doi: 10.1377/hlthaff.2019.01835, PMID: 32897793

[18] Gordon SH Hoagland A Admon LK Daw JR Lindsay BU Admon K. Extended postpartum medicaid eligibility is associated with improved continuity of coverage in the postpartum year. Health Aff. (2022) 41:69–78. doi: 10.1377/hlthaff.2021.00730 34982627

[19] Yarmuth JA (2021). Text—H.R.1319—117th congress (2021-2022): American rescue plan act of 2021. Available at: https://www.congress.gov/bill/117th-congress/house-bill/1319/text

[20] KFF (2022). Postpartum coverage extension in the American rescue plan act of 2021. Available at: https://www.kff.org/policy-watch/postpartum-coverage-extension-in-the-american-rescue-plan-act-of-2021/ (Accessed Sep 12, 2022).

[21] AHCCCS (n.d.). Medical policy manual (AMPM). Available at: https://www.azahcccs.gov/shared/MedicalPolicyManual/

[22] KIDS COUNT Data Center (2023). Births covered by AHCCCS (Medicaid). Available at: https://datacenter.kidscount.org/data/tables/191-births-covered-by-ahcccs-medicaid#detailed/2/any/false/37,871,870,573,869,36,868,867,133,38/any/10539

[23] Office of the Arizona Governor (2023). Securing Arizona’s future: governor ducey signs fiscal year 2023 budget. Available at: https://azgovernor.gov/governor/news/2022/06/securing-arizonas-future-governor-ducey-signs-fiscal-year-2023-budget

[24] Arizona Health Care Cost Containment System (AHCCCS) (2022). Reference subsystem codes and values 2022. Available at: https://www.azahcccs.gov/PlansProviders/Downloads/CodesValues.pdf

[25] CDC (2022). Pregnancy-related deaths: data from maternal mortality review committees in 36 US states, 2017–2019. Available at: https://www.cdc.gov/reproductivehealth/maternal-mortality/erase-mm/data-mmrc.html

[26] Center for Health Statistics N (2022). Vital statistics reporting guidance a reference guide for certification of deaths associated with pregnancy on death certificates.

[27] AmeriHealth Caritas Delaware (2021). HEDIS ® adult 2021 documentation and coding guidelines effectiveness of care: prevention and screening measure/coding tips measure description documentation required coding breast cancer screening (BCS). Available at: www.ncqa.org/publications (Accessed October 21, 2022).

[28] USDA ERS (n.d.). Rural-urban commuting area codes. Available at: https://www.ers.usda.gov/data-products/rural-urban-commuting-area-codes.aspx

[29] HRSA (2022). Defining Rural Population. Available at: https://www.hrsa.gov/rural-health/about-us/what-is-rural

[30] Desisto CL Rohan A Handler A Saria AS Johnson T. The effect of continuous versus pregnancy-only Medicaid eligibility on routine postpartum Care in Wisconsin, 2011-2015. 1234567890. Matern Child Health J. (2020) 24:1138–50. doi: 10.1007/s10995-020-02924-4, PMID: 32335806

[31] Rankin KM Haider S Caskey R Chakraborty A Roesch P Handler A. Healthcare utilization in the postpartum period among Illinois women with Medicaid paid claims for delivery, 2009-2010. Matern Child Health J. (2016) 20:144–53. doi: 10.1007/s10995-016-2043-8 27339649 PMC5290055

[32] Berman DR (2019). Revisiting the urban-rural relationship in Arizona. Available at: https://morrisoninstitute.asu.edu/sites/default/files/urban-rural_relationship.pdf

[33] KFF (2022). Federal Medical Assistance Percentage (FMAP) for Medicaid and Multiplier. Available at: https://www.kff.org/medicaid/state-indicator/federal-matching-rate-and-multiplier/?currentTimeframe=0&sortModel=%7B%22colId%22:%22Location%22,%22sort%22:%22asc%22%7D 10297498

[34] KFF (2021). Expanding Postpartum Medicaid Coverage. Available at: https://www.kff.org/womens-health-policy/issue-brief/expanding-postpartum-medicaid-coverage/

[35] Tyree PT Lind BK Lafferty WE. Challenges of using medical insurance claims data for utilization analysis. Am J Med Qual. (2006) 21:269–75. doi: 10.1177/1062860606288774 16849784 PMC1533763

[36] Essien UR Molina RL Lasser KE. Strengthening the postpartum transition of care to address racial disparities in maternal health. J Natl Med Assoc. (2019) 111:349–51. doi: 10.1016/j.jnma.2018.10.016 30503575

[37] Kozhimannil KB Vogelsang CA Hardeman RR Prasad S. Disrupting the pathways of social determinants of health: doula support during pregnancy and childbirth. J Am Board Fam Med. (2016) 29:308–17. doi: 10.3122/jabfm.2016.03.150300 27170788 PMC5544529

