# Supplementary material for: Extended Medicaid coverage will improve access but insufficient to enhance postpartum care utilization: a secondary analysis of the 2016–2019 Arizona Medicaid claims
Source: Front Public Health. 2024 Jan 8;11:1281574. doi: 10.3389/fpubh.2023.1281574 (PMC10801226; doi:10.3389/fpubh.2023.1281574)
Supplement: Supplementary file 1 [file Table_1.DOCX]

Supplementary Material

Extended Medicaid Coverage Will Improve Access but Insufficient to Enhance Postpartum Care Utilization: A Secondary Analysis of the 2016 - 2019 Arizona Medicaid Claims.

Abidemi Okechukwu^1*^, Ivo Abraham^2^, Chinedu Okechukwu^3^, Priscilla Magrath^1^, David G. Marrero^4^, Leslie V. Farland^1^, Halimatou Alaofe^1^

*** Correspondence:** Abidemi Okechukwu [aokechukwu@arizona.edu](mailto:aokechukwu@arizona.edu)

**SUPPLEMENTARY TABLES**

**Supplementary Table 1.0 Insurance Eligibility Category Codes**

| **Inclusion Codes** | | **Exclusion Codes** |
| --- | --- | --- |
| Pregnancy-related insurance | Continuous Insurance | Emergency services for Medicaid non-eligible women & services for incarcerated women |
| "361" "360" "368" "464" | 231, 232, 140, 586, 589, 275, 588, 332, 334, 410, 422, 415, 490, 955, 953. | 900, 950, 905, 908, ST1, 060, 210, 220, 350, 352, 351, 357, 305, 260, 270, 265, 462, 560. |

**Supplementary Table 2.0. Diagnosis Codes to determine the types of postpartum service.**

| **Category of Service** | **Diagnosis Codes (ICD-10, HCPCS, HPT)** |
| --- | --- |
| Childbirth Encounter | O80, 082 |
| Routine Postpartum visit | 57170, 58300, 59430, 99501,0503F, Z01411, Z01419, 59618, 5962”, 59400, 59410, 59510, 59515, 59610, 59614 |
| Cervical Cytology | 88141, 88142, 88143, 88147, 88148, 88150, 88152, 88153, 88154, 88164,88165, 88166, 88167, 88174, 88175, Z0142, Z430430, Z391, Z392, G0123, G0124, G0141, G0143, G0144, G0145, G0147, G0148, P3000, P3001, Q0091 |
| Complications of Puerperium | O860,O8600, O8601, O8602, O8603, O8604, O8609, O8611, O8612, O8613, O8619,O8620, O8621, O8622, O8629, O864, O8681,O8689, 0870, 0871 ,O872, O873, O874, O878, O879, O88011, O88012, O88013, O8803, O88111, O88112, O88113, O88119, O8812, O8813, O882, O882, O88211, O88212 ,O88213, O88219 ,O8822, O8823, O88312, O88313, O8833, O88512 ,O88811, O88812, O88813, O88819, O8882, O8883, O8901, O8909, O891,O892, O894, O895, O898, O899, O900, O901, O902, O903, O904, O905 ,O906, O9081 ,O9089 ,O909, O91012, O91013, O91019, O9102, O9103, O91111, O91112, O91113, O91119 ,O9112, O9113, O91211, O91212, O91213, O91219, O9122 ,O9123 ,O9181, O92011, O92012, O92013, O9202, O9203, O92111, O92112, O92113, O92119, O9212, O9213, O9220 , O9229, O923 ,O924, O925, O926, O9270 ,O9279, O924, O925 ,O926 ,O9270 |
| Contraception and Family Planning (LARCS) | J7297, J7298, J7300, J7301, J7307 |
| Medication-Assisted Treatment (MAT) | H2010, H0020 |
| Emergency Procedures | 59409, 59514, 59612, 59620 |

**Supplementary Table 3.0 Test of Interaction by Type of Delivery Route and Residence in the Association between Postpartum Visit and Insurance Type**

|  | **Adjusted Model ^a^** | |
| --- | --- | --- |
|  | Odds ratio | (95% CI) |
| **Type of Delivery Route** |  |  |
| Cesarean Section | 0.82 | 0.65, 1.04 |
| Vaginal Delivery | 0.69 | 0.66, 0.74 |
| Log-likelihood test for interaction (p-value)^b^ |  | 0.63 |
| **Type of Residence** |  |  |
| Rural | 0.71 | 0.67, 0.76 |
| Urban | 0.61 | 0.52, 0.73 |
| Log-likelihood test for interaction (p-value) | 0.50 | |
| Abbreviations: 95% CI, 95 percent confidence interval  ^a^ Models were adjusted for age, race, prenatal visit, and complications at childbirth.  ^b^ p-value less than 0.05 for the interaction was considered significant | | |

**Supplementary Table 4. Poisson Regression Model for Factors Associated with the Counts of Postpartum Visits**

| **Characteristic** | **Poisson Regression Estimate** | **Standard Error** | **Adjusted Incidence Risk Ratios** |
| --- | --- | --- | --- |
| **Insurance Type** |  |  |  |
| Pregnancy-related Insurance | -0.1610 | 0.01367 | 0.85127 |
| Continuous Insurance | 0 [Reference] | . | 1.00000 |
| **Age** |  |  |  |
| 21-29 | 0.08489 | 0.01879 | 1.08860 |
| 30-39 | 0.1333 | 0.01955 | 1.14254 |
| 40-49 | 0.1689 | 0.03027 | 1.18405 |
| 18-20 | 0 [Reference] | . | 1.00000 |
| **Race/Ethnicity** |  |  |  |
| Asian | 0.05889 | 0.02735 | 1.06065 |
| Black/African American | 0.008515 | 0.01250 | 1.00855 |
| Hispanic | -0.05658 | 0.01336 | 0.94499 |
| Native American | 0.07397 | 0.02106 | 1.07677 |
| Other^a^ | -0.07003 | 0.05824 | 0.93236 |
| Pacific Islander | -0.02059 | 0.01808 | 0.97962 |
| White | 0 [Reference] | . | 1.00000 |
| **Type of residence** |  |  |  |
| Rural | -0.09171 | 0.01371 | 0.91237 |
| Urban | 0 [Reference] | . | 1.00000 |
| **Prenatal visit** |  |  |  |
| Yes | -0.1629 | 0.02609 | 0.84971 |
| No | 0 [Reference] | . | 1.00000 |
| **Route of delivery** |  |  |  |
| Vaginal | 0.6118 | 0.008559 | 1.84368 |
| Cesarean | 0 [Reference] | . | 1.00000 |
| **Maternal morbidity after childbirth** |  |  |  |
| Yes | 0.2336 | 0.02964 | 1.26317 |
| No | 0 [Reference] | . | 1.00000 |

^a^ Women who identified as multiple races or ethnicities.

**Supplementary Table 5.0.A: C-statistic: Test of Model Discrimination**

| **Association of Predicted Probabilities and Observed Responses** | | | |
| --- | --- | --- | --- |
| Percent Concordant | 60.4 | Somers' D | 0.308 |
| Percent Discordant | 29.6 | Gamma | 0.342 |
| Percent Tied | 10.0 | Tau-a | 0.135 |
| Pairs | 747101180 | c | 0.654 |

**Supplementary Table 5.0.B: Hosmer and Lemeshow Goodness-of-Fit Test**

| **Hosmer and Lemeshow Goodness-of-Fit Test** | | |
| --- | --- | --- |
| Chi-Square | DF | Pr > ChiSq |
| 8.7394 | 7 | 0.2719 |
| A chi-square p-value less than 0.05 for the interaction was considered significant for non-fit. | | |
